# Decompressive hemicraniectomy after aneurysmal subarachnoid hemorrhage—justifiable in light of long-term outcome?

**DOI:** 10.1007/s00701-022-05250-6

**Published:** 2022-05-21

**Authors:** Michael Veldeman, Miriam Weiss, Lorina Daleiden, Walid Albanna, Henna Schulze-Steinen, Omid Nikoubashman, Hans Clusmann, Anke Hoellig, Gerrit Alexander Schubert

**Affiliations:** 1 Department of Neurosurgery, RWTH Aachen University Hospital, Pauwelsstrasse 30, 52074 Aachen, Germany; 2 Department of Neurosurgery, Kantonsspital Aarau, Aarau, Switzerland; 3 Department of Neurosurgery, Military Hospital Koblenz, Koblenz, Germany; 4 Department of Intensive Care Medicine, RWTH Aachen University, Aachen, Germany; 5 Department of Diagnostic and Interventional Neuroradiology, RWTH Aachen University, Aachen, Germany

**Keywords:** Subarachnoid hemorrhage, Decompressive hemicraniectomy, Extended Glasgow outcome scale, Delayed cerebral ischemia

## Abstract

**Purpose:**

Decompressive hemicraniectomy (DHC) is a potentially lifesaving procedure in refractory intracranial hypertension, which can prevent death from brainstem herniation but may cause survival in a disabled state. The spectrum of indications is expanding, and we present long-term results in a series of patients suffering from aneurysmal subarachnoid hemorrhage (SAH).

**Methods:**

We performed a retrospective analysis of previously registered data including all patients treated for SAH between 2010 and 2018 in a single institution. Patients treated with decompressive hemicraniectomy due to refractory intracranial hypertension were identified. Clinical outcome was assessed by means of the Glasgow outcome scale after 12 months.

**Results:**

Of all 341 SAH cases, a total of 82 (24.0%) developed intracranial hypertension. Of those, 63 (18.5%) patients progressed into refractory ICP elevation and were treated with DHC. Younger age (OR 0.959, 95% CI 0.933 to 0.984; *p* = 0.002), anterior aneurysm location (OR 0.253, 95% CI 0.080 to 0.799; 0.019; *p* = 0.019), larger aneurysm size (OR 1.106, 95% CI 1.025 to 1.194; *p* = 0.010), and higher Hunt and Hess grading (OR 1.944, 95% CI 1.431 to 2.641; *p* < 0.001) were independently associated with the need for DHC. After 1 year, 10 (15.9%) patients after DHC were categorized as favorable outcome. Only younger age was independently associated with favorable outcome (OR 0.968 95% CI 0.951 to 0.986; *p* = 0.001).

**Conclusions:**

Decompressive hemicraniectomy, though lifesaving, has only a limited probability of survival in a clinically favorable condition. We identified young age to be the sole independent predictor of favorable outcome after DHC in SAH.

**Supplementary Information:**

The online version contains supplementary material available at 10.1007/s00701-022-05250-6.

## Introduction

Decompressive hemicraniectomy (DHC) is a potentially lifesaving surgical procedure as it can effectively alleviate intracranial pressure (ICP) elevation refractory to medical management. Although originally described by Kocher and Cushing in the early twentieth century, the procedure rapidly fell into disgrace due to the high complication rate and its limited effect on outcome [15]. Fueled by promising results in the context of malignant cerebral ischemia [8, 13, 18] and traumatic brain injury [3, 9] decompressive hemicraniectomy has now firmly cemented itself in the treatment algorithms of these disease entities and may also be beneficial in selected patients with aneurysmal subarachnoid hemorrhage (SAH) [1, 6, 12, 16].

Decision for DHC warrants prior assessment of anticipated clinical outcome. By preventing brainstem herniation and resulting death, the procedure allows patients to survive despite possible severe cerebral damage. The dreaded effect of DHC might be a tradeoff, with reduced mortality but higher rate of patients surviving in a vegetative state. In malignant stroke, the clinical effect of territorial ischemia is predictable to some extent and can be assessed prior to surgery and discussed with the patient’s next of kin. In subarachnoid hemorrhage, patients remain at risk of further secondary damage, even after DHC. Secondary damage occurs in the form of so-called delayed cerebral ischemia (DCI). The occurrence of DCI-related infarction has been identified as one of the main outcome-defining factors in subarachnoid hemorrhage [21]. In recent literature, there is a broader acceptance of DHC in poor-grade SAH patients based on increased survival and rates of favorable outcome in around one-fourth of patients. Identifying early predictors of ICP decompensation enables to consider early or even prophylactic decompression. Unfortunately, existing series provide insufficient insight into the occurrence of DCI, DCI-related infarction, and the time point in relation to decompressive surgery.

The goal of this analysis is to assess clinical outcome after decompressive hemicraniectomy, to identify factors predictive of refractory intracranial hypertension, and to identify factors associated with favorable outcome after DHC.

## Material and methods

### Patient population

This study is part of an observational cohort study, of which data have been partially published previously [19, 20]. Previously published analyses focused on the effect of introduction of invasive monitoring for DCI detection. The incentive for this analysis was driven by an internal quality control assessing complications and outcome after decompressive hemicraniectomy in our SAH patient collective. The data collection process was part of a previously registered observational study (NCT02142166) and was approved by the local ethics committee of the Medical Faculty of RWTH Aachen University (EK 062/14). Prospectively included patients or their legal representative signed informed consent.

All consecutive SAH cases, presented between 2010 and 2018 in a single university hospital, were considered for inclusion. The causal aneurysm needed to be identified in either CT- or conventional cerebral angiography, and patients aged 18 and 90 years were included. Patients where early mortality was anticipated (mydriatic pupil for > 45 min., other signs of brainstem herniation, or global cerebral ischemia) were excluded.

### Standard treatment

After diagnosis of SAH, the offending aneurysm was secured within 48 h via either surgical clipping or endovascular occlusion (coiling, flow-diverter stenting, or WEB-device placement). All patients were equipped with central venous access and blood pressure was monitored continuously via an arterial line. In case of acute hydrocephalus, an external ventricular drain was placed prior to aneurysm occlusion. Patients were thereafter surveilled on our neurointensive care unit for a minimum of 14 days. After aneurysm occlusion, patients received an initial wake-up test and were treated with prophylactic oral nimodipine.

### Intracranial pressure measurement and management

In patients with an external ventricular drain, ICP was measured intermittently. Patients failing an initial wake-up test were considered for placement of invasive neuromonitoring to assist in detection of delayed cerebral ischemia (DCI), consisting of combined ICP and brain tissue oxygen pressure (p_ti_O_2_) measurement as well as cerebral microdialysis (CMD) (Neurovent PTO, Raumedic, Helmbrechts, Germany; 71 High Cut-Off Brain Microdialysis Catheter, μdialysis, Stockholm, Sweden). Elevated ICP above 20 mmHg, if not associated with a wake-up attempt and improving neurological status, was treated via a standing institutional algorithm. After optimization of cerebral venous outflow by head elevation of 30° and providing sufficient cerebral perfusion pressure, sedation was started consisting of first tier midazolam (up to 0.3 mg/kg/h) combined with sufentanil (up to 0.3 mg/kg/h). Second-tier treatment included clonidine (up to 12 mg/h) and ketamine (up to 0.4 mg/kg/h), and third-tier allowed the addition of propofol infusion (up to 50 µg/kg/h). Bolus administration of hyperosmolar agents (mannitol 15%; 0.5–1 g/kg) and barbiturates (thiopental; 1–2 mg/kg) was used only as a bridging therapy during CT transports or transportation to the operating room. Neuromuscular blocking agents were avoided and only applied if respiratory distress was identified as a contributing factor to ICP elevation. Hyperventilation was reserved for patients with ICP decompensation on their way to the operating room and during preparation for decompressive surgery.

Refractory ICP elevation was defined as ICP persisting above 20 mmHg for 15 min or longer, not controllable via the above-mentioned treatments and after exclusion of other causes, e.g., ventilation problems, EVD obstruction, or aberrant measurement. In case of sudden ICP decompensation, emergency CT was performed to rule out hemorrhagic complications or other causal factors with direct effect on further treatment decisions.

As last-tier treatment, a large unilateral frontotemporoparietal decompressive hemicraniectomy in rapid closure technique without duraplasty was considered [7, 23]. Whenever possible, the dominant hemisphere was decompressed unless space-occupying bleeding or infarction prompted non-dominant decompression. Minimum anteroposterior diameter of the bone flap of 12 cm was guaranteed [17]. Decompressive surgery performed concomitant to aneurysm closure was coined as primary DHC, whereas all DCH thereafter was considered secondary.

### Delayed cerebral ischemia

Delayed cerebral ischemia was diagnosed clinically whenever possible (new focal neurologic deficit or a decrease in GCS ≥ 2 for at least 1 h, not ascribable to other diagnoses) [22]. In unconscious patients, DCI surveillance was achieved by either repeated perfusion CT-imaging or invasive neuromonitoring including brain tissue oxygen monitoring, cerebral microdialysis, or both. Our detailed DCI diagnostic and treatment algorithm has been described previously [19, 20].

First-tier DCI treatment consisted of induced euvolemic arterial hypertension by infusion of intravenous norepinephrine. Endovascular rescue treatment (ERT) was considered in patients without clinical or radiological improvement, persisting hypoperfusion, or anaerobic metabolism. Endovascular treatment included balloon angioplasty for localized proximal vessel narrowing or spasmolysis with intra-arterial nimodipine for diffuse distal vasospasm.

### Cranioplasty

All surviving patients received their cryopreserved (− 80 °C) autologous bone flap. The time point of cranioplasty depended on the development of their rehabilitation process; however, early (< 30 days) cranioplasty was avoided to prevent infection and CSF fistula [14].

### Outcome definition

The primary endpoint was defined as favorable outcome in SAH patients after DHC. Clinical outcome was assessed by means of the extended Glasgow outcome scale (GOS-E) at discharge, and after 6 as well as 12 months, and was dichotomized into unfavorable (GOS-E_1-4_) and favorable (GOS-E_5-8_) outcomes. A blinded assessor prospectively collected outcome data in a structured telephone interview. In patients included prior to 2014, outcome data was collected during regular follow-ups and missing information was appended by analysis of patient files.

### Statistical analysis

All data are presented as mean and standard deviation for normally and as median and interquartile range for non-normally distributed continuous variables. Categorical variables are provided as frequencies and percentages. After normality testing via the Shapiro–Wilk test and plotting, the appropriate statistical test was selected. For nominal data, the chi-square test was used; for normally distributed continuous data, the independent sample t-test and for non-normally distributed data the Mann–Whitney *U*-test were used. Factors affecting the need for DHC and occurrence of favorable outcome were assessed in a logistic regression model. Predictor covariates were included when univariable results presented with *p* < 0.15. Linearity of continuous or ordinal variables with respect to the logit of the dependent variable was assessed via the Box-Tidwell procedure. A Bonferroni correction was applied to adjust the *p* value cut-off using all terms in the model. All statistical analyses were performed using IBM SPSS Statistics 25 (SPSS Inc., Chicago, IL, USA) and graphics were plotted using GraphPad Prism 9.0.1 (GraphPad Software, Inc., La Jolla, CA, USA). Statistical significance was defined as a two-sided *p* < 0.05.

## Results

### Study population

**Fig. 1:**
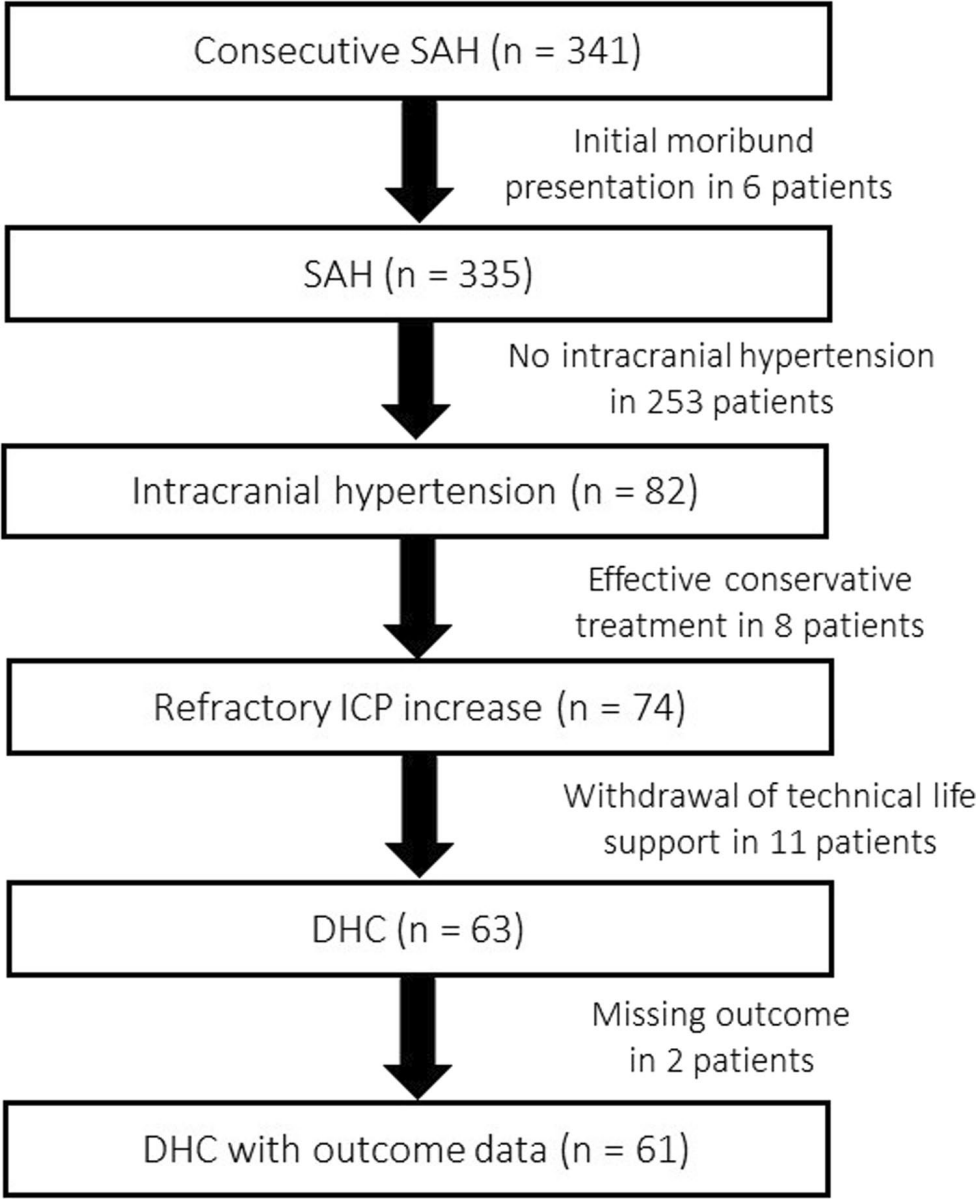
Flow-chart of patient recruitment. DHC, decompressive hemicraniectomy; SAH, subarachnoid hemorrhage

During the inclusion period, 341 consecutive SAH patients were treated at our institution. Six patients were excluded due to early mortality. Of the remaining 335 patients, 82 (24.5%) suffered increased intracranial pressure (ICP > 20). Thereof, eight (9.8%) patients were managed successfully with conservative treatment. Of the 74 patients with refractory ICP increase (ICP > 20; > 15 min.), 11 (14.9%) were not treated with DHC due to anticipated poor quality of life based on comorbidity and/or dominant hemispheric and/or brainstem damage on imaging studies. Subsequently, in accordance with patient’s will, life support was withdrawn. The remaining 63 (85.1%) patients who developed refractory ICP were treated via DHC. An inclusion flow-chart is provided as Fig. 1.

### ICP increase in SAH

When comparing SAH patients developing ICP increase, patients with higher-grade hemorrhages according to the Hunt and Hess and modified Fisher scale suffered from ICP increase, more often. Additionally, larger aneurysm size (7.0 ± 5.0 mm *vs.* 6.0 ± 4.0 mm; *p* = 0.017) and aneurysms of the anterior circulation (89.0% *vs.* 75.5%; *p* = 0.009) were associated with ICP increase in univariate comparison. Relevant baseline data is provided in Table 1.

**Table 1:** Baseline characteristics of the entire SAH cohort and comparison between patients developing increased intracranial pressure or not

	All (*n* = 335)	No ICP increase (*n* = 253)	ICP increase (*n* = 82)	*p* value
**Demographics**				
Age—years; mean ± SD	55.1 ± 13.1	56.0 ± 13.4 (23–90)	52.1 ± 10.5 (19–72)	**0.053**
Sex—female/**male; no. (%)**	233 (69.6)/102 (30.4)	173 (68.4) / 80 (31.6)	60 (73.2)/22 (26.8)	0.413
**Comorbidity**				
Hypertension; no. (%)	144 (43.0)	114 (45.2)	30 (36.6)	0.169
Smoking; no. (%)	108 (32.2)	83 (32.9)	25 (30.5)	0.681
Diabetes; no. (%)	14 (4.2)	13 (5.2)	1 (1.2)	0.122
BMI; median (IQR)	25.0 (6)	25 (5)	25 (6)	0.607
**Aneurysm location; no. (%)**				**0.003**
Acomm	100 (30.0)	83 (32.8)	17 (20.7)	
MCA	96 (28.7)	58 (22.9)	38 (46.3)	
ICA	43 (12.8)	28 (11.1)	15 (18.3)	
Others	96 (28.7)	84 (33.2)	12 (14.6)	
Ant. circulation	264 (78.8)	191 (75.5)	73 (89.0)	**0.009**
Post. circulation	71 (21.1)	62 (24.5)	9 (11.0)	
**Aneurysm size** Max. diameter (mm)—mean ± SD	6.0 ± 4.0	6.0 ± 4.0	7.0 ± 5.0	**0.017**
**Hemorrhage severity**				
**Hunt and Hess grade; no. (%)**				** < 0.001**
Grade 1	65 (19.4)	61 (24.1)	4 (4.9)	
Grade 2	80 (23.9)	68 (26.9)	12 (14.6)	
Grade 3	96 (28.7)	75 (29.6)	21 (25.6)	
Grade 4	53 (15.8)	28 (11.1)	25 (30.5)	
Grade 5	41 (12.2)	21 (8.3)	20 (24.4)	
**Modified Fisher scale; no. (%)**				** < 0.001**
Grade 1	82 (24.5)	76 (30.0)	6 (7.3)	
Grade 2	46 (13.7)	39 (15.4)	7 (8.5)	
Grade 3	98 (29.3)	72 (28.5)	26 (31.7)	
Grade 4	109 (32.5)	66 (26.1)	43 (52.4)	
Acute hydrocephalus; no. (%)	218 (65.1)	154 (61.1)	64 (78.0)	**0.005**
**Aneurysm occlusion; no. (%)**				
Clipping/endovascular	152 (45.4)/183 (54.6)	106 (41.9)/147 (58.1)	46 (56.1)/36 (43.9)	**0.025**
**Invasive neuromonitoring (p**_**ti**_**O**_**2**_**/CMD); no. (%)**	99 (29.6)	59 (23.3)	40 (48.8)	** < 0.001**

*Acomm* anterior communication artery; *BMI* body mass index; *DHC* decompressive hemicraniectomy; *ICA* internal carotid artery; *MCA* middle cerebral artery; *SAH* subarachnoid hemorrhage

### ICP decompensation and the decision to perform DHC

Treatment of ICP began a median of 2 days (IQR 3.0) after SAH, and conservative ICP treatment was successful in 8 (9.8%) patients. Of all 74 patients with ICP decompensation, 11 patients were not treated with DHC because of concomitant septic shock in 2 cases, multiple organ failure in 3 cases, 1 case of dominant-side rebleeding, and 4 cases of dominant or bilateral DCI-related cerebral infarction. These patients were on average 10 years older compared to surgically treated patients (60.5 ± 13.6 *vs.* 50.7 ± 10.9 years of age; *p* = 0.010). In the remainder of 63 (85.1%) patients with refractory ICP increase, DHC was performed a median of 2 days (IQR 4.0) after aneurysm rupture (Suppl. Tables 1 and 2).

### Early factors associated with the decision to perform DHC

When comparing patient who were treated via DHC versus patients who were not, age, aneurysm location and maximum diameter, and Hunt and Hess and modified Fisher grading along aneurysm occlusion modality were identified as cofactor associated with DHC. These factors were introduced into the logistic regression model (Table 2). Of these six factors, four were independently associated with the need for DHC: younger age (OR 0.959, 95%CI 0.933 to 0.984; *p* = 0.002), anterior circulation aneurysm location (OR 0.253, 95% CI 0.080 to 0.799; 0.019), increasing aneurysm size (OR 1.106, 95% CI 1.025 to 1.194), and increasing Hunt and Hess grading (OR 1.944, 95% CI 1.431 to 2.641; *p* < 0.001) (Suppl. Table 3).

**Table 2:** Baseline characteristics of all subarachnoid hemorrhage patients and univariate comparison between patients with or without decompressive hemicraniectomy

	All (*n* = 335)	no DHC (*n* = 272)	DHC (*n* = 63)	*p* value
**Demographics**				
Age—years;- mean ± SD	55.1 ± 13.1	56.2 ± 13.3	50.7 ± 10.9	**0.002**
Sex—female/male; no. (%)	233 (69.6)/102 (30.4)	187 (68.8)/85 (31.3)	46 (73.0)/17 (27.0)	0.507
**Comorbidity**				
Hypertension; no. (%)	144 (43.0)	121 (44.5)	23 (36.5)	0.240
Smoking; no. (%)	108 (32.2)	89 (32.7)	19 (30.2)	0.682
Diabetes; no. (%)	14 (4.2)	13 (4.8)	1 (1.6)	0.252
BMI; median (IQR)	25.0 (6)	25.2 (6)	25.0 (6)	0.622
**Aneurysm location; no. (%)**				** < 0.001**
Acomm	100 (30.0)	88 (32.4)	12 (19.0)	
MCA	96 (28.7)	62 (22.8)	34 (54.0)	
ICA	43 (12.8)	31 (11.4)	12 (19.0)	
Others	96 (28.7)	91 (33.5)	5 (7.9)	
Ant. circulation	264 (78.8)	205 (75.4)	59 (93.7)	**0.001**
Post. circulation	71 (21.1)	67 (24.6)	4 (6.3)	
**Aneurysm size** Max. diameter max. (mm) ± SD	6.0 ± 4.0	6.0 ± 4.0	7.0 ± 5.0	**0.013**
**Hemorrhage severity**				
**Hunt and Hess grade; no. (%)**				** < 0.001**
Grade 1	65 (19.4)	62 (22.8)	3 (4.8)	
Grade 2	80 (23.9)	71 (26.1)	9 (14.3)	
Grade 3	96 (28.7)	83 (30.5)	13 (20.6)	
Grade 4	53 (15.8)	31 (11.4)	22 (34.9)	
Grade 5	41 (12.2)	25 (9.2)	16 (25.4)	
**Modified Fisher scale; no. (%)**				** < 0.001**
Grade 1	82 (24.5)	76 (27.9)	6 (9.5)	
Grade 2	46 (13.7)	43 (15.8)	3 (4.8)	
Grade 3	98 (29.3)	76 (27.9)	22 (34.9)	
Grade 4	109 (32.5)	77 (28.3)	32 (50.8)	
Acute hydrocephalus; no. (%)	218 (65.1)	172 (63.2)	46 (73.0)	0.152
**Aneurysm occlusion; no. (%)**				
Clipping/endovascular	152 (45.4)/183 (54.6)	114 (41.9)/158 (58.1)	38 (60.3)/25 (39.7)	**0.008**

*Acomm* anterior communication artery; *BMI* body mass index; *DHC* decompressive hemicraniectomy; *ICA* internal carotid artery; *MCA* middle cerebral artery; *SAH* subarachnoid hemorrhage

### Clinical outcome after DHC

**Fig. 2:**
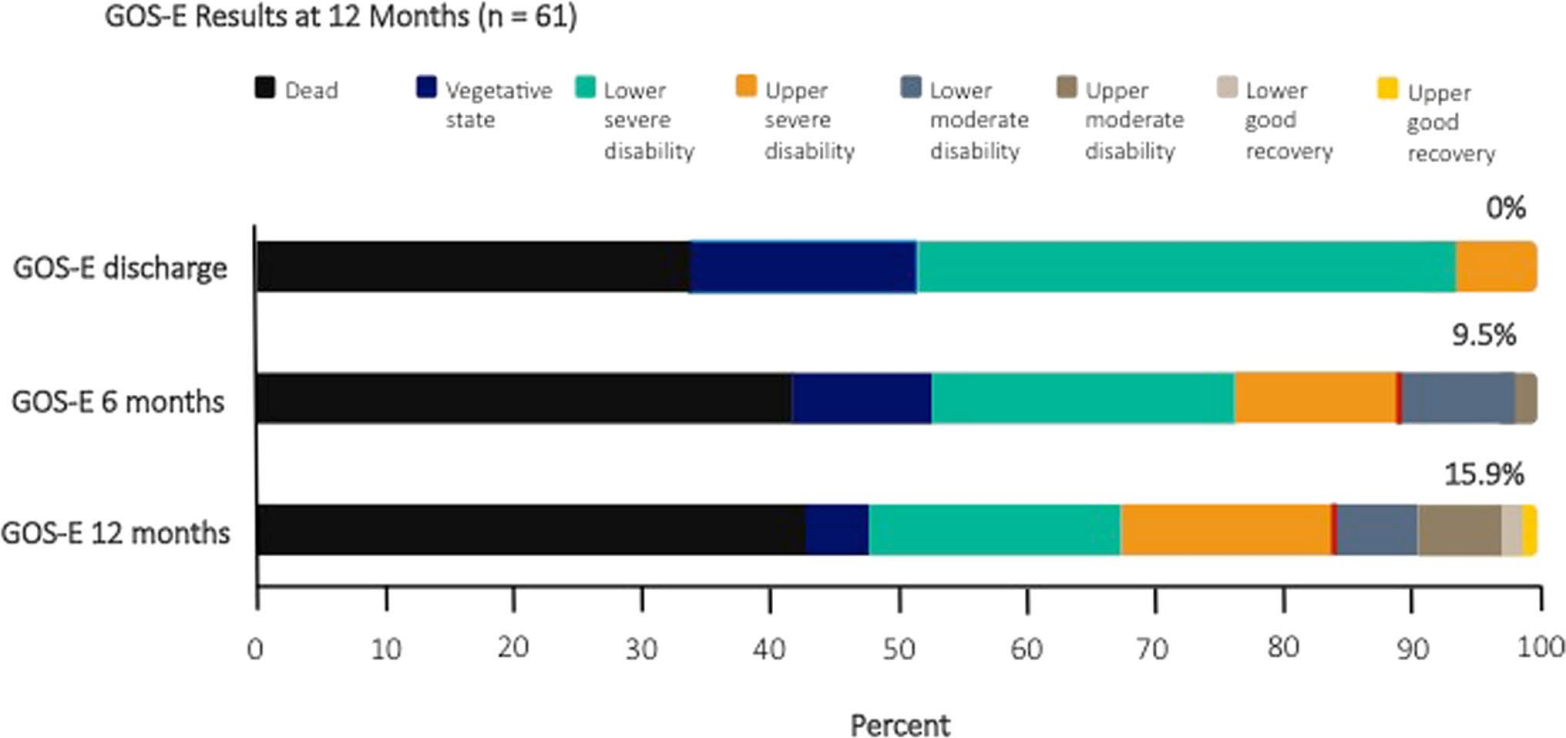
Stacked bar charts of outcome as measured via the extended Glasgow outcome scale (GOS-E) at discharge, after 6 and after 12 months in 61 SAH patients treated with decompressive hemicraniectomy. GOS-E, the extended Glasgow outcome scale; SAH, aneurysmal subarachnoid hemorrhage

No patient with DHC was discharged with favorable outcome. After 1 year, 10 (15.9%) patients with DHC had reached favorable outcome. Outcome grading according to the extended Glasgow outcome scale and shifts over time are visually depicted in Fig. 2. Only age and aneurysm occlusion modality were identified as covariates to be included in a logistic regression model. Of both, only younger age was independently associated with favorable outcome GOS-E_5-8_ (OR 0.968 95% CI 0.951 to 0.986; *p* = 0.001) (Table 3).

**Table 3:** Overview of logistic regression results assessing the effects of covariates on the decision for decompressive hemicraniectomy and the occurrence of favorable outcome and vegetative state

	no DHC (*n* = 272)	DHC (*n* = 63)	OR	95% CI	*p* value
Age—yrs.—mean ± SD	56.2 ± 13.3	50.7 ± 10.9	0.958	0.933 to 0.984	**0.002**
Aneurysm location	See Suppl. Table 1	See Suppl. Table 1	0.253	0.080 to 0.799	**0.019**
Aneurysm size max. diameter (mm) ± SD	6.0 ± 4.0	7.0 ± 5.0	1.106	1.025 to 1.194	**0.010**
Hunt and Hess grade	See Table 2	See Table 2	1.944	1.431 to 2.641	** < 0.001**
Modified Fisher scale	See Table 2	See Table 2	1.394	0.987 to 1.968	0.059
Aneurysm occlusion modality	See Table 2	See Table 2	0.535	0.277 to 1.035	0.063
	Favorable outcome (*n* = 10)	Unfavorable outcome (*n* = 51)	OR	95% CI	*p* value
Age—yrs.—mean ± SD	43.3 ± 11.9	51.8 ± 10.1	0.968	0.951 to 0.986	**0.001**
Occlusion modality (clipping / endovascular)	8 (80.0) / 2 (20.0)	28 (54.9) / 23 (45.1)	1.026	0.641 to 1.644	0.914
	Vegetative state (*n* = 11)	GOS-E ≥ 3 (*n* = 31)	OR	95% CI	*p* value
ICH	6 (54.5)	26 (83.9)	0.556	0.092 to 3.352	0.522
Aneurysm occlusion modality	See Table 2	See Table 2	0.111	0.020 to 0.614	**0.012**

*CI* confidence interval; *DHC* decompressive hemicraniectomy; *ICH* intracerebral hemorrhage; *OR* odds ratio; *SD* standard deviation

### Vegetative state

At discharge, 11 (17.5%) patients treated with DHC were in vegetative state (GOS-E grade 2). Of those, 3 (4.8%) patients remained in vegetative state after 1 year, 5 (7.9%) patients had died, and 3 patients (4.8%) reached a state of lower severe disability being bedridden and dependent of daily assistance. The presence of an intracerebral hematoma and the aneurysm occlusion modality were included into a logistic regression model. Surgical aneurysm occlusion was independently associated with a lower risk of vegetative state (OR 0.111, 95% CI 0.020 to 0.614; *p* = 0.012) (Table 3).

### Multimodal monitoring

**Fig. 3:**
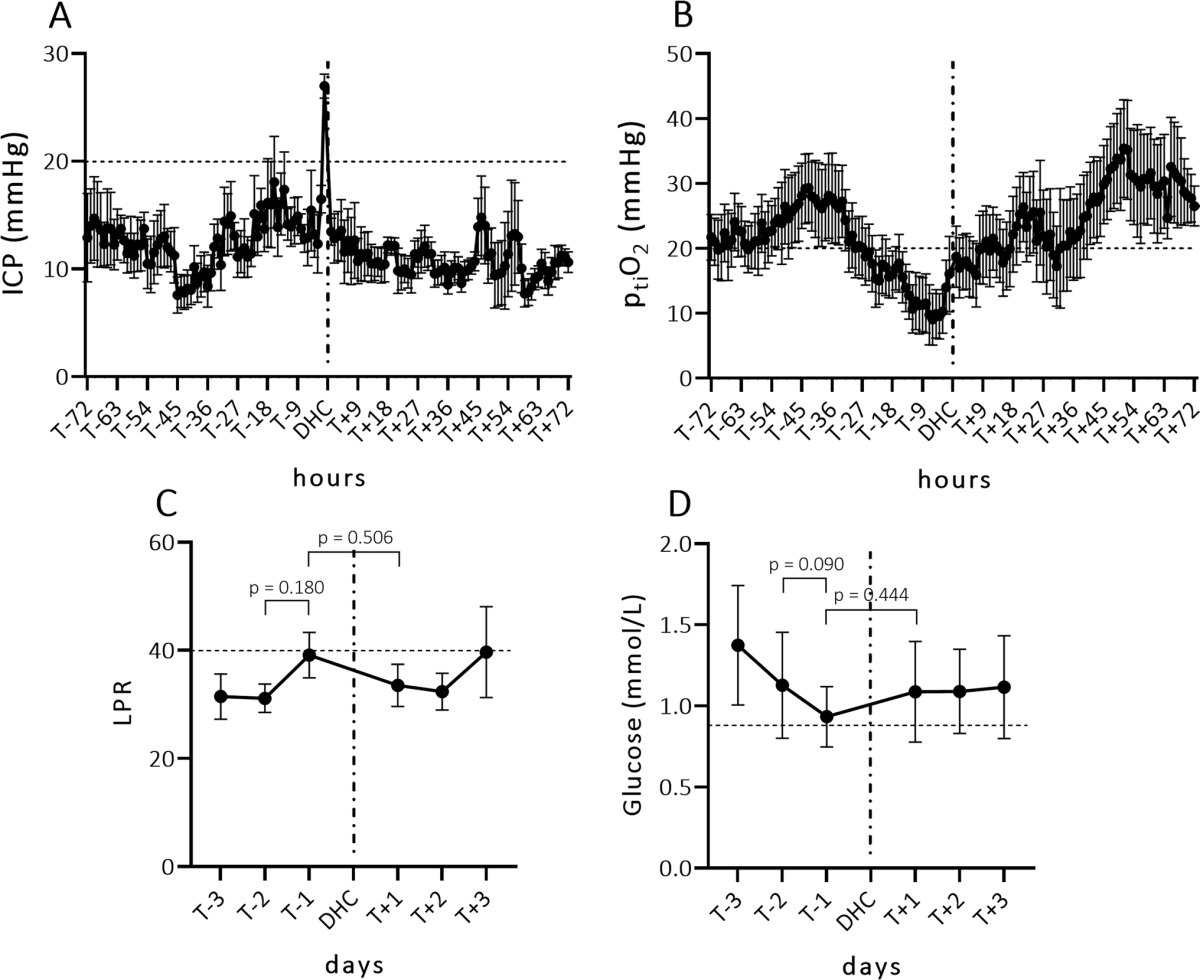
Course of invasively measured data presented as means and standard deviation, in patients who suffered aneurysmal subarachnoid hemorrhage and were treated for refractory intracranial pressure with decompressive hemicraniectomy (*n* = 22). **A** Course of intracranial pressure (mean ± SD) 3 days prior and 3 days after DHC. **B** Course of brain tissue oxygen pressure (mean ± SD) 3 days prior and 3 days after DHC. **C** Course of the lactate to pyruvate ratio (mean ± SD) 3 days prior and 3 days after DHC. **D** Course of interstitial glucose levels (mean ± SD) as measured in CMD 3 days prior and 3 days after DHC. The pathological cut-off is depicted as a horizontal dashed line for each individual parameter. ICP, intracranial pressure; DHC, decompressive hemicraniectomy; LPR, lactate to pyruvate ratio; ptiO_2_, brain tissue oxygen pressure; SD, standard deviation

In 22 patients, multimodal neuromonitoring (p_ti_O_2_/CMD) was available 48 h prior to DHC. Data of the first 24 h after placement was not used due to an initial calibration phase. Of this subgroup of patients, the course of ICP and p_ti_O_2_ around the DHC procedure is plotted in Fig. 3. Intracranial pressure did not increase gradually before decompressive surgery. Typically, medical treatment was able to keep ICP within normal range, after which unannounced rapid decompensations prompted surgery (Fig. 3A). When looking at p_ti_O_2_ data prior to DHC, a gradual decrease of oxygen pressure was observed 48 h before DHC (Fig. 3B).

Lactate to pyruvate ratio (LPR) and glucose measurements obtained via microdialysis were averaged over 24 h to account for fluctuations in the frequency of data acquisition. When looking at plotted daily average LPR, the ratio tends to go up before refractory ICP occurs; however, changes leading up to DHC as well as in a before and after comparison, were not significantly different (Fig. 3C). When plotted, mean glucose levels tend to drop in the days preceding ICP decompensation. No significant difference, however, was noted comparing the two days prior to DHC or before and after DHC (Fig. 3D).

### Primary DHC versus secondary DHC

The number of patients in our cohort treated with primary DHC in this series was comparatively low (*n* = 8; 13.1%) When comparing patient- and hemorrhage-specific characteristics between patients undergoing primary DHC *vs.* secondary DHC, no relevant differences were noted. No differences in clinical or radiological SAH severity were identified. After 1 year, 2 (25.0%) patients who underwent primary DHC had died *versus* 26 (42.6%) patients treated with secondary DHC. After 1 year, 2 (25.0%) patients reached favorable outcome after primary DHC and 8 (15.1%) patients after secondary DHC (*p* = 0.481) (Suppl. Table 3).

## Discussion

In our series of subarachnoid hemorrhage patients treated with decompressive hemicraniectomy, younger age, aneurysms of the anterior circulation, larger aneurysm diameter, and higher Hunt and Hess grading were independent predictors of impending ICP decompensation. When looking at clinical outcome after 1 year, 43.1% of patients with DHC had died and 15.9% achieved favorable outcome. Younger age was identified as the single predictor variable independently associated with more favorable outcome. As a secondary endpoint, we aimed at identifying factors contributing to patients remaining in a vegetative state. Not clearly explicable, clip occlusion of the causal aneurysm was independently associated with a lower risk of vegetative state. A possible explanation could be the confounding higher rate of clip occlusion in patients with predominantly intracerebral hematoma and therefore a lower risk of DCI and DCI-related infarction (Table 4).

**Table 4:** Comparison of patient- and hemorrhage-specific factors between dichotomized outcome groups as measured by the extended Glasgow outcome scale after 12 months

	All DHC with outcome data (*n* = 61)	Favorable outcome (*n* = 10)	Unfavorable outcome (*n* = 51)	*p* value
**Demographics**				
Age—years; mean ± SD	50.6 ± 10.9	43.3 ± 11.9	51.8 ± 10.1	**0.024**
Sex–female/male	45 (73.8)/16 (26.2)	8 (72.7)/2 (18.2)	37 (77.1)/14 (29.2)	0.624
**ICP crisis**				
Lag ICP crisis (days) (IQR)	2.0 (3.0)	1.0 (3.0)	2.0 (5.0)	0.501
Lag DHC (days) (IQR)	2.0 (4.0)	3.0 (3.0)	2.0 (5.0)	0.836
Primary/secondary DHC	8 (13.1)/53 (86.9)	2 (20.0)/8 (80.0)	6 (11.8)/45 (88.2)	0.481
**Aneurysm location; no. (%)**				0.524
Ant. circulation	59 (96.7)	10 (100.0)	49 (96.1)	
Post. circulation	2 (3.3)	0 (0.0)	2 (3.9)	
**Aneurysm size** Max. diameter (mm)—mean ± SD	7.0 ± 5.0	7.5 ± 9.0	7.0 ± 5.0	0.506
**Hemorrhage severity**				
**Hunt and Hess grade; no. (%)**				0.908
Grade 1	3 (4.9)	0 (0.0)	3 (5.9)	
Grade 2	9 (14.8)	2 (20.0)	7 (13.7)	
Grade 3	14 (23.0)	2 (20.0)	12 (23.5)	
Grade 4	21 (34.4)	4 (40.0)	17 (33.3)	
Grade 5	14 (23.0)	2 (20.0)	12 (23.5)	
**Modified Fisher scale; no. (%)**				0.433
Grade 1	6 (98.4)	2 (20.0)	4 (7.8)	
Grade 2	36 (59.0)	1 (10.0)	2 (3.9)	
Grade 3	22 (36.1)	2 (20.0)	20 (39.2)	
Grade 4	30 (49.2)	5 (50.0)	25 (49.0)	
**Aneurysm occlusion; no. (%)**				**0.140**
Clipping/endovascular	28 (45.9)/23 (37.7)	8 (80.0)/2 (20.0)	28 (54.9)/23 (45.1)	
**DCI treatment; no. (%)**				
DCI	44 (72.1)	7 (70.0)	37 (72.5)	0.869
ERT	25 (41.0)	4 (40.0)	21 (41.2)	0.945
Spasmolysis	22 (36.1)	3 (30.0)	19 (37.3)	0.262
CLINA	7 (11.5)	2 (20.0)	5 (9.8)	0.355
**Clinical outcome; no. (%)**				
DCI-related infarction	28 (45.9)	3 (30.0)	25 (49.0)	0.270
**Cranioplasty**	34 (55.7)	10 (100.0)	24 (47.1)	
Lag (days)	85.0 (84.0)	93.5 (84.0)	70.0 (63.0)	0.512
Early (≤ 90)/late (> 90)	13 (21.3)/21 (34.4)	5 (50.0)/5 (50.0)	8 (33.3)/16 (31.4)	

*CLINA* continuous local intra-arterial nimodipine applications; *DHC* decompressive hemicraniectomy; *ERT* endovascular rescue treatment; *ICP* intracranial pressure; *IQR* interquartile range; *SD* standard deviations

Most published series of DHC after SAH report a lower rate of unfavorable outcome after early DHC [2, 12]. Early identification of patients with high risk of ICP decompensation is therefore mandatory. An additional argument in favor of early decompression rising from our series would be the high rate of refractory ICP increase (74 out of 82; 90.2%). Once ICP increases, the chance of ICP elevation not sufficiently responsive to medical treatment alone is high, and we acknowledge the value of identifying patients at high risk of ICP decompensation adequately and early. Jabbarli et al. identified intracerebral hemorrhage, “rapid” vasospasm on angiography, early cerebral infarction, aneurysm diameter larger than 5 mm, clipping, age under 55, Hunt and Hess grade 4 or 5, reduced consciousness, and the presence of an external ventricular drain as risk factors associated with ICP decompensation and the need for decompressive hemicraniectomy [10].

In a limited subgroup, we identified that gradual drop in brain tissue oxygen tension precedes ICP decompensation. From our limited data of patients with invasive neuromonitoring prior to ICP decompensation, brain tissue oxygen tension might prove to be a useful additive parameter to foresee ICP increase.

Darkwah Oppong et al. included 28 observational studies in their comprehensive meta-analysis and identified younger age and good initial clinical state as the most relevant independent factors contributing to favorable outcome [4]. In a further exploration of their own series, larger surface area of the removed bone flap was independently associated with a lower risk of cerebral infarction and unfavorable outcome [11]. In another meta-analysis, Alotaibi et al. combined 15 DHC studies in SAH patients, and identified an overall rate of poor outcome of 61.2% and death of 27.8% after 12 months [1]. The authors concluded here that mainly the lack of robust control groups limits the conclusions that can be drawn from published series.

**Fig. 4:**
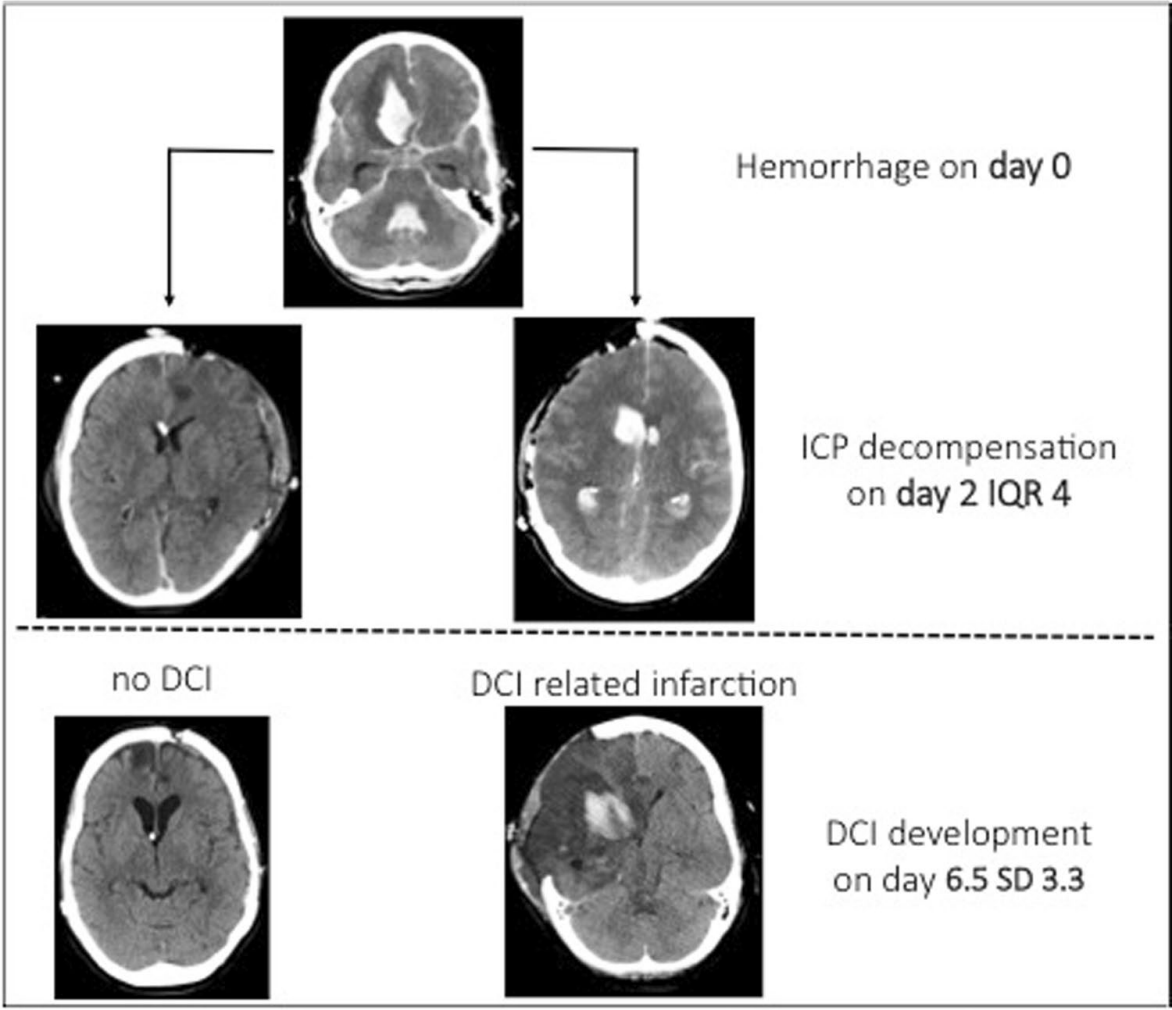
Depiction of the timely relation between decompensation of intracranial pressure warranting decompressive hemicraniectomy and the development of delayed cerebral ischemia in subarachnoid hemorrhage. DCI, delayed cerebral ischemia; IQR, interquartile range

Existing observational studies have not reported rates of DCI and DCI-related infarction after DCH in SAH. In our series, the median time point of ICP decompensation preceded the first diagnosed DCI event by somewhat more than four days (Fig. 4). Moreover, factors associated with ICP decompensation and risk of DCI largely overlap, i.e., clinical and radiological severity, age, and aneurysm size. This means that at the time point of ICP decompensation and DHC indication, the effects of DCI have to be taken into account in relation to patient’s recovery.

Dorfer et al. stratified their series of decompressed SAH patients based on the pathology underlying intracranial hypertension and observed better outcome results with DHC performed for ICP increase caused by parenchymal hematoma compared to decompression for cerebral edema or DCI-related infarction [5]. Although initially attempted, we refrained from etiologically classifying the reasons for ICP decompensation, as multiple pathophysiological processes, e.g., intraparenchymal bleeding, focal edema around the bleeding, infarction development, and generalized cerebral edema oftentimes coincide in a single patient.

To date, malignant stoke remains the only level A evidence for prophylactic decompressive hemicraniectomy. The anticipated neurological deficit patients will carry after the acute phase are anticipatable in case of territorial cerebral infarction. In traumatic brain injury and subarachnoid hemorrhage, the sequelae of secondary damage yet to come remain unpredictable in nature. Results of randomized TBI trials regarding DHC [3, 9] are still controversially discussed, and the reason thereof being the necessity to question the practice of resolving a mechanical issue despite severe underlying disease carrying the risk of surviving in a vegetative state. For SAH, only a randomized trial with dichotomization at the point of exhausted medical ICP management into surgical (DHC) or continued medical treatment arm—in analogy to TBI trials—could shed light on this problem. It is the personal opinion of the authors that vegetative state is an unwanted result from an ethical, socio-economic, and personal standpoint as it can be considered a prolonging of human suffering. This perception of, and dealing with the risk of vegetative state is, however, heterogeneous and deeply rooted in the cultural and religious background of patients and treating medical personnel.

### Limitations

Obvious limitations of this analysis are the observational design and lack of a control group. Outcome data was partly retrospectively collected, and two patients were lost to follow-up of which no clinical outcome beyond discharge is available and unfavorable outcome or even death of these patients is plausible. In contrast to other DHC series, we did not limit the analysis to poor-grade SAH cases. This was decided to better reflect the subarachnoid hemorrhage population and increase extrapolation of results. Our ICP treatment protocol is not tailored towards SAH patients, as it is also applied to patients with TBI. There exist no SAH-specific ICP treatment guidelines, and to the best of our knowledge, no studies comparing different medical treatment algorithms have been performed so far.

## Conclusion

Decompressive hemicraniectomy in patients suffering aneurysmal subarachnoid hemorrhage remains a controversial topic. In our series, rates of mortality and unfavorable outcome remain fairly high. Selection of patients remains crucial, and only younger age is identified as an independent factor associated with more favorable outcome. In the majority of cases, decompensation of intracranial pressure precedes delayed cerebral ischemia and the risk of further secondary damage.

## Supplementary Information

Below is the link to the electronic supplementary material.

Supplementary file1 (DTA 47 KB)

## Data Availability

The raw data of this analysis can be made available by the authors to any qualified researcher.
