# Supplementary material for: Decompressive hemicraniectomy after aneurysmal subarachnoid hemorrhage—justifiable in light of long-term outcome?
Source: Acta Neurochir (Wien). 2022 May 21;164(7):1815–26. doi: 10.1007/s00701-022-05250-6 (PMC9233638; doi:10.1007/s00701-022-05250-6)
Supplement: Supplementary file 1 — Supplementary file1 (DTA 47 KB) [file 701_2022_5250_MOESM1_ESM.docx]

**Supplemental Table 1**

|  | **Effective ICP treatment (n = 8)** | **ICP decompensation (n = 74)** | **p-value** |
| --- | --- | --- | --- |
| **Demographics** |  |  |  |
| Age - yrs. - mean ± SD | 57.3 ± 7.6 (47 - 69) | 52.1 ± 11.8 (19 - 84) | 0.231 |
| Sex - Female / Male | 5 (62.5) / 3 (37.5) | 55 (74.3) / 19 (25.7) | 0.473 |
| **Comorbidity** |  |  |  |
| Hypertension | 2 (25.0) | 28 (37.8) | 0.474 |
| Smoking | 2 (25.0) | 23 (31.1) | 0.723 |
| Diabetes | 0 (0) | 1 (1.4) | 0.741 |
| BMI - median (IQR) | 24 (5) | 25 (6) | 0.541 |
| **Aneurysm location - no. (%)** |  |  | **< 0.001** |
| Acomm | 0 (0) | 17 (23.0) |  |
| MCA | 2 (25.0) | 36 (48.6) |  |
| ICA | 1 (12.5) | 14 (18.9) |  |
| Others | 5 (6.3) | 7 (9.5) |  |
| Ant. circulation | 5 (62.5) | 68 (91.9) | **0.012** |
| Post. circulation | 3 (37.5) | 6 (8.1) |  |
| **Aneurysm size**  Max. diameter (mm) - mean ± SD | 6.5 ± 5.0 | 7.0 ± 5.0 | 0.833 |
| **Hemorrhage severity** |  |  |  |
| **Hunt and Hess grade - no. (%)** |  |  | 0.236 |
| Grade 1 | 0 (0) | 4 (5.4) |  |
| Grade 2 | 3 (37.5) | 9 (12.2) |  |
| Grade 3 | 3 (37.5) | 18 (24.3) |  |
| Grade 4 | 1 (12.5) | 24 (32.4) |  |
| Grade 5 | 1 (12.5) | 19 (25.7) |  |
| **Modified Fisher scale - no. (%)** |  |  | 0.775 |
| Grade 1 | 0 (0) | 6 (8.1) |  |
| Grade 2 | 1 (12.5) | 6 (8.1) |  |
| Grade 3 | 2 (25.0) | 24 (32.4) |  |
| Grade 4 | 5 (62.5) | 38 (51.4) |  |
| Acute hydrocephalus | 6 (75.0) | 55 (74.3) | 0.967 |
| **Aneurysm occlusion - no. (%)** |  |  |  |
| Clipping / Endovascular | 3 (37.5) / 5 (62.5) | 43 (58.1) / 31 (41.9) | 0.256 |

**Suppl. Table 1.** Comparison of subarachnoid hemorrhage patients with intracranial pressure increase and effective conservative treatment versus intracranial pressure increase refractory to conservative treatment.

Acomm, anterior communication artery; BMI, body mass index; DHC, decompressive hemicraniectomy; ICA, internal carotid artery; MCA, middle cerebral artery; SAH, subarachnoid hemorrhage; SD, standard deviation.

**Supplemental Table 2**

|  | **WOLS (n = 11)** | **DHC (n = 63)** | **p-value** |
| --- | --- | --- | --- |
| **Demographics** |  |  |  |
| Age - yrs. - mean ± SD | 60.5 ± 13.6 | 50.7 ± 10.9 | **0.010** |
| Sex - Female / Male | 9 (91.8) / 2 (18.2) | 46 (73.0) / 17 (27.0) | 0.537 |
| **Comorbidity** |  |  |  |
| Hypertension | 5 (45.5) | 23 (36.5) | 0.572 |
| Smoking | 4 (36.4) | 19 (30.2) | 0.682 |
| Diabetes | 0 (0) | 1 (1.6) | 0.674 |
| BMI - median (IQR) | 26 (6) | 25.0 (6) | 0.440 |
| **Aneurysm location - no. (%)** |  |  | 0.090 |
| Acomm | 5 (45.5>) | 12 (19.0) |  |
| MCA | 2 (18.2) | 34 (54.0) |  |
| ICA | 2 (18.2) | 12 (19.0) |  |
| Others | 2 (18.2) | 5 (7.9) |  |
| Ant. circulation | 9 (81.8) | 59 (93.7) | 0.185 |
| Post. circulation | 2 (18.2) | 4 (6.3) |  |
| **Aneurysm size**  Max. diameter (mm) - mean ± SD | 7.0 ± 2.0 | 7.0 ± 5.0 | 0.464 |
| **Hemorrhage severity** |  |  |  |
| **Hunt and Hess grade - no. (%)** |  |  | 0.278 |
| Grade 1 | 1 (9.1) | 3 (4.8) |  |
| Grade 2 | 0 (0) | 9 (14.3) |  |
| Grade 3 | 5 (45.5) | 13 (20.6) |  |
| Grade 4 | 2 (18.2) | 22 (34.9) |  |
| Grade 5 | 3 (27.3) | 16 (25.4) |  |
| **Modified Fisher scale - no. (%)** |  |  | 0.052 |
| Grade 1 | 0 (0) | 6 (9.5) |  |
| Grade 2 | 3 (27.3) | 3 (4.8) |  |
| Grade 3 | 2 (18.2) | 22 (34.9) |  |
| Grade 4 | 6 (54.5) | 32 (50.8) |  |
| Acute hydrocephalus | 10 (90.9) | 45 (71.4) | 0.172 |
| **Aneurysm occlusion - no. (%)** |  |  |  |
| Clipping / Endovascular | 5 (45.5) / 6 (54.5) | 38 (60.3) / 25 (39.7) | 0.357 |

**Suppl. Table 2.** Comparison of SAH patients with ICP decompensation treated with decompressive hemicraniectomy or withdrawal of technical life support.

Acomm, anterior communication artery; BMI, body mass index; DHC, decompressive hemicraniectomy; ICA, internal carotid artery; MCA, middle cerebral artery; SAH, subarachnoid hemorrhage; WOLS, withdrawal of technical life support.

**Supplemental Table 3.**

|  | **Primary DHC (n = 8)** | **Secondary DHC (n = 53)** | **p-value** |
| --- | --- | --- | --- |
| **Demographics** |  |  |  |
| Age - yrs. - mean ± SD (range) | 53.8 ± 6.2 | 50.1 ± 11.4 | 0.382 |
| Sex - Female / Male | 7 (87.5) / 1 (12.5) | 39 (73.6) / 14 (26.4) | 0.323 |
| Lag ICP crisis (days) (IQR) | 0.0 (0) | 2.0 (4.0) | **< 0.001** |
| **Aneurysm location - no. (%)** |  |  | 0.929 |
| Acomm | 1 (12.5) | 11 (20.8) |  |
| MCA | 6 (75.0) | 28 (52.8) |  |
| ICA | 1 (12.5) | 11 (20.8) |  |
| Others | 0 (0) | 3 (5.7) |  |
| Ant. circulation | 8 (100.0) | 52 (98.1) | 0.498 |
| Post. circulation | 0 (0) | 3 (1.9) |  |
| **Aneurysm size**  Max. diameter (mm) - mean ± SD | 7.5 ± 16 | 7.0 ± 5.0 | 0.335 |
| **Hemorrhage severity** |  |  |  |
| **Hunt and Hess grade - no. (%)** |  |  | 0.671 |
| Grade 1 | 0 (0) | 3 (5.7) |  |
| Grade 2 | 0 (0) | 9 (17.0) |  |
| Grade 3 | 2 (25.0) | 12 (22.6) |  |
| Grade 4 | 3 (37.5) | 18 (34.0) |  |
| Grade 5 | 3 (37.5) | 13 (24.5) |  |
| **Modified Fisher scale - no. (%)** |  |  | 0.470 |
| Grade 1 | 0 (0) | 6 (11.3) |  |
| Grade 2 | 1 (12.5) | 2 (11.3) |  |
| Grade 3 | 2 (25.0) | 20 (37.7) |  |
| Grade 4 | 5 (62.5) | 27 (50.9) |  |
| **Aneurysm occlusion - no. (%)** |  |  | 0.817 |
| Clipping / Endovascular | 5 (62.5) / 3 (37.5) | 32 (60.4) /23 (43.4) |  |
| **GOS-E 12 months** |  |  | **0.038** |
| Dead | 2 (25.0) | 26 (42.6) |  |
| Vegetative state | 0 (0) | 4 (6.6) |  |
| Lower severe disability | 4 (50.0) | 12 (19.7) |  |
| Upper sever disability | 0 (0) | 9 (14.8) |  |
| Lower moderate disability | 1 (12.5) | 4 (6.6) |  |
| Upper moderate disability | 0 (0) | 4 (6.6) |  |
| Lower good recovery | 0 (0) | 1 (1.6) |  |
| Upper good recovery | 1 (12.5) | 1 (1.6) |  |
| **GOS-E 12 months – dichotomized** |  |  | 0.481 |
| Unfavorable outcome | 6 (75.0) | 45 (84.9) |  |
| Favorable outcome | 2 (25.0) | 8 (15.1) |  |

**Suppl. Table 3.**

Acomm, anterior communication artery; DHC, decompressive hemicraniectomy; ICA, internal carotid artery; ICP, intracranial pressure; MCA, middle cerebral artery.
